# Evolution of Resistance to Targeted Anti-Cancer Therapies during Continuous and Pulsed Administration Strategies

**DOI:** 10.1371/journal.pcbi.1000557

**Published:** 2009-11-06

**Authors:** Jasmine Foo, Franziska Michor

**Affiliations:** Computational Biology Program, Memorial Sloan-Kettering Cancer Center, New York, New York, United States of America; Tufts University, United States of America

## Abstract

The discovery of small molecules targeted to specific oncogenic pathways has revolutionized anti-cancer therapy. However, such therapy often fails due to the evolution of acquired resistance. One long-standing question in clinical cancer research is the identification of optimum therapeutic administration strategies so that the risk of resistance is minimized. In this paper, we investigate optimal drug dosing schedules to prevent, or at least delay, the emergence of resistance. We design and analyze a stochastic mathematical model describing the evolutionary dynamics of a tumor cell population during therapy. We consider drug resistance emerging due to a single (epi)genetic alteration and calculate the probability of resistance arising during specific dosing strategies. We then optimize treatment protocols such that the risk of resistance is minimal while considering drug toxicity and side effects as constraints. Our methodology can be used to identify optimum drug administration schedules to avoid resistance conferred by one (epi)genetic alteration for any cancer and treatment type.

## Author Summary

Recently, the field of anti-cancer therapy has witnessed a revolution by the discovery of targeted therapy, which refers to compounds targeting specific pathways causing abnormal growth of cancer cells. The clinical success of such drugs has been limited by the evolution of acquired resistance to these compounds, which leads to a relapse after initial response to therapy. Current dosing procedures are not designed to optimally delay the emergence of resistance; the identification of such optimal dosing schedules represents an important challenge in clinical cancer research. Here, we design a novel methodology to identify the optimum drug administration strategies that reach this clinical goal. Our model describes the evolutionary dynamics of a tumor cell population during therapy. We consider drug resistance emerging due to a single (epi)genetic alteration and calculate the probability of resistance arising during specific dosing strategies. We then optimize treatment protocols such that the risk of resistance is minimal while considering drug toxicity and side effects as constraints. Since this methodology can be extended to describe situations arising during administration of cytotoxic chemotherapy as well, it can be used to identify optimum drug administration schedules to avoid resistance for any cancer and treatment type.

## Introduction

Alteration of the normal regulation of cell-cycle progression, division and death lies at the heart of the processes driving tumorigenesis. A detailed molecular understanding of these processes provides an opportunity to design targeted anti-cancer agents. The term ‘targeted therapy’ refers to drugs with a focused mechanism that specifically act on well-defined protein targets or biological pathways that, when altered by therapy, impair the abnormal proliferation of cancer cells. Examples of this type of therapy include hormonal-based therapies in breast and prostate cancer; small-molecule inhibitors of the EGFR pathway in lung, breast, and colorectal cancers – such as erlotinib (Tarceva), gefitinib (Iressa), and cetuximab (Erbitux); inhibitors of the JAK2, FLT3 and BCR-ABL tyrosine kinases in leukemias – such as imatinib (Gleevec), dasatinib (Sprycel), and nilotinib (Tasigna); blockers of invasion and metastasis; anti-angiogenesis agents like bevacizumab (Avastin); proapoptotic drugs; and proteasome inhibitors such as bortezomib (Velcade) [1],[2]. The target-driven approach to drug development contrasts with the conventional, more empirical approach used to develop cytotoxic chemotherapeutics, and the successes of the past few years illustrate the power of this concept. The absence of prolonged clinical responses in many cases, however, stresses the importance of continued basic studies into the mechanisms of targeted drugs and their failure in the clinic.

Acquired drug resistance is an important reason for the failure of targeted therapies. Resistance emerges due to drug metabolism, drug export, and alteration of the drug target by mutation, deletion, or overexpression. Depending on the cancer type and its stage, the therapy administered, and the genetic background of the patient, one or several (epi)genetic alterations may be necessary to confer drug resistance to cells. In this paper, we investigate drug resistance emerging due to a single alteration. For example, treatment of chronic myeloid leukemia (CML) with the targeted agent imatinib fails due to acquired point mutations in the BCR-ABL kinase domain [3]. To date, ninety different point mutations have been identified, each of which is sufficient to confer resistance to imatinib [4]. The second-generation BCR-ABL inhibitors, dasatinib and nilotinib, can circumvent most mutations that confer resistance to imatinib; the T315I mutation, however, causes resistance to all BCR-ABL kinase inhibitors developed so far. Similarly, the T790M point mutation in the epidermal growth factor receptor (EGFR) confers resistance to the EGFR tyrosine kinase inhibitors gefitinib and erlotinib [5], which are used to treat non-small cell lung cancer. Other mechanisms of resistance include gene amplification or overexpression of the P-glycoprotein family of membrane transporters (e.g., MDR1, MRP, LRP) which decreases intracellular drug accumulation, changes in cellular proteins involved in detoxification or activation of the drug, changes in molecules involved in DNA repair, activation of oncogenes such as Her-2/Neu, c-Myc, and Ras as well as inactivation of tumor suppressor genes like p53 [6]–[11].

The design of optimal drug administration schedules to minimize the risk of resistance represents an important issue in clinical cancer research. Currently, many targeted drugs are administered continuously at sufficiently low doses so that no drug holidays are necessary to limit the side effects. Alternatively, the drug may be administered at higher doses in short pulses followed by rest periods to allow for recovery from toxicity. Clinical studies evaluating the advantages of different approaches have been ambivalent. Some investigations found that a low-dose continuous strategy is more effective [12], while others have advocated more concentrated dosages [13]. The effectiveness of a low-dose continuous approach is often attributed to its targeted effect on tumor endothelial cells and the prevention of angiogenesis rather than low rates of resistance [14]. The continuous dosing strategy is often implemented as combination therapy, sometimes including a second drug administered at a higher dose in a pulsed fashion.

A significant amount of research effort has been devoted to developing mathematical models of tumor growth and response to chemotherapy. In a seminal paper, Norton and Simon proposed a model of kinetic (not mutation-driven) resistance to cell-cycle specific therapy in which tumor growth followed a Gompertzian law [15]. The authors used a differential equation model in which the rate of cell kill was proportional to the rate of growth for an unperturbed tumor of a given size. They suggested that one way of combating the slowing rate of tumor regression was to increase the intensity of treatment as the tumor became smaller, thus increasing the chance of cure. The authors also published a review of clinical trials employing dosing schedules related to their proposed dose-intensification strategy, and concluded that the concept of intensification was clinically feasible, and possibly efficacious [16]. Later, predictions of an extension of this model were validated in a clinical trial evaluating the effects of a dose-dense strategy and a conventional regimen for chemotherapy [17]. Their model and its predictions have become known as the Norton-Simon hypothesis and have generated substantial interest in mathematical modeling of chemotherapy and kinetic resistance. For example, Dibrov and colleagues formulated a kinetic cell-cycle model to describe cell synchronization by cycle phase-specific blockers [18]; this model was then used for optimizing treatment schedules to increase the degree of synchronization and thus the effectiveness of a cycle-specific drug. Agur introduced another model describing cell-cycle dynamics of tumor and host cells to investigate the effect of drug scheduling on responsiveness to chemotherapy [19]; this model was then used to optimize scheduling of chemotherapeutics to maximize efficacy while controlling host toxicity. Other theoretical studies include a mathematical model of tumor recurrence and metastasis during periodically pulsed chemotherapy [20], a control theoretic approach to optimal dosing strategies [21], and an evaluation of chemotherapeutic strategies in light of their anti-angiogenic effects [14]. For a more comprehensive survey of kinetic models of tumor response to chemotherapy, we refer the reader to reviews [22]–[24] and references therein.

There have also been substantial research efforts devoted to developing mathematical models of genetic resistance, i.e. resistance driven by genetic alterations in cancer cells. Since mutations conferring resistance can arise as random events during the DNA replication phase of cell division, the dynamics of resistant populations are well-suited to description with stochastic mathematical models. Coldman and co-authors pioneered this field by introducing stochastic models of resistance against chemotherapy to guide treatment schedules (see e.g., [25],[26] and references therein). In 1986, Coldman and Goldie studied the emergence of resistance to one or two functionally equivalent chemotherapeutic drugs using a branching process model of tumor growth with a differentiation hierarchy [25]. In this model, the birth and death rates of cells were time-independent constants and each sensitive cell division gave rise to a resistant cell with a certain probability. The effect of drug was modeled as an additional probabilistic cell kill law on the existing population, and the drug could be administered in a fixed dose at a series of fixed time points. The goal of the model was to schedule the sequential administration of both drugs in order to maximize the probability of cure. Later, the assumption of equivalence or ‘symmetry’ between the two drugs was relaxed [27]. These models were also extended to include the toxic effects of chemotherapy on normal tissue, and an optimal control problem was formulated to maximize the probability of tumor cure without toxicity [26]. More recently, Iwasa and colleagues used a multi-type birth-death process model to study the probability of resistance emerging due to one or multiple mutations in populations under selection pressure [28]. The authors considered pre-existing resistance mutations and determined the optimum intervention strategy utilizing multiple drugs. Multi-drug resistance was also investigated using a multi-type birth-death process model in work by Komarova and Wodarz [29],[30]. In their models, the resistance to each drug was conferred by genetic alterations within a mutational network. The birth and death rates of each cell type were time-independent constants and cells had an additional drug-induced death rate if they were sensitive to one or more of the drugs. The authors studied the evolution of resistant cells both before and after the start of treatment, and calculated the probability of treatment success under continuous treatment scenarios with a variable number of drugs. Recently, the dynamics of resistance emerging due to one or two genetic alterations in a clonally expanding population of sensitive cells prior to the start of therapy were studied using a time-homogenous multi-type birth-death process [31],[32].

One common feature of these models of genetic resistance is that the treatment effect is formulated as an additional probabilistic cell death rate on sensitive cells, separate from the underlying birth and death process model with constant birth and death rates. Under these model assumptions, the drug cannot alter the proliferation rate of either sensitive or resistant cells; however, a main effect of many targeted therapies (e.g. imatinib, erlotinib, gefinitib) is the inhibition of proliferation of cancer cells. Inhibited proliferation in turn leads to a reduced probability of resistance since resistant cells are generated during sensitive cell divisions. In this paper, we utilize a non-homogenous multi-type birth-death process model wherein the birth and death rates of both sensitive and resistant cells are dependent on a temporally varying drug concentration profile. This study represents a significant departure from existing models of resistance since we incorporate the effect of inhibition of sensitive cell proliferation as well as drug-induced death, obtaining a more accurate description of the evolutionary dynamics of the system. In addition, we generalize our model to incorporate partial resistance, so that the drug may also have an effect on the birth and death rates of resistant cells. The goals of our analysis also differ from those of previous work. Coldman and Murray were interested in finding the optimal administration strategy for multiple chemotherapeutic drugs in combination or sequential administration [26]; they aimed to maximize the probability of cure while limiting toxicity. Komarova was interested in studying the effect of multiple drugs administered continuously on the probability of eventual cure [30]. In contrast, in this paper we derive estimates for the expected size of the resistant cell population as well as the probability of resistance during a full spectrum of continuous and pulsed treatment schedules with one targeted drug. We then propose a methodology for selecting the optimal strategy from this spectrum to minimize the probability of resistance as well as to maximally delay the progression of disease by controlling the expected size of the resistant population, while incorporating toxicity constraints. In many clinical scenarios, the probability of resistance is high regardless of dosing strategy, and thus the maximal delay of disease progression is a more realistic objective than tumor cure. The methodology developed in this paper can be applied to study acquired resistance in any cancer and treatment type.

## Methods

Consider a population of 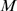 drug-sensitive cancer cells at the time of diagnosis. These cells may represent the total bulk of tumor cells or, alternatively, cancer stem cells only; if only the latter cells are capable of self-renewal to produce and maintain a resistant cell clone, then the effective population size 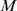 equals the number of cancer stem cells in the tumor. We assume for now that there are no resistant cells present at the time of diagnosis; this assumption will be relaxed in later sections. The evolutionary dynamics of the sensitive and resistant cancer cell populations are modeled as a multi-type branching process [33].

Let us first consider this process during a continuous dosing schedule. Cells are chosen at random to divide or die in accordance with a specified growth and death rate. During therapy, the growth and death rates of sensitive cancer cells are denoted by 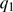 and 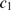, respectively. During each sensitive cancer cell division, a resistant cell arises with probability 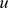; such a cell has growth and death rates 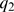 and 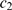 during treatment. The growth and death rates during therapy are determined by the dose intensity of treatment.

Let 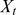 denote the random process representing the number of sensitive cancer cells at time 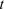, and let 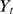 denote the number of resistant cancer cells at time 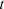. The initial conditions at the start of treatment, 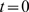, are defined as 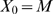 and 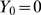. Denote the state of the process after the 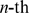 event (represented by a cell birth or death) as 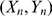. The transition probabilities of the stochastic process are given by
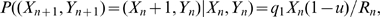


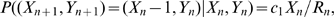


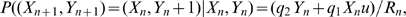


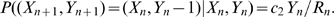
Here 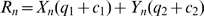 is the sum of the rates, normalizing the total probability to 1. The arrival times between events are exponentially distributed: if 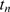 denotes the time at the beginning of the 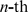 step, then 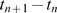 is an exponential random variable with parameter 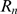.

**Figure 1:**
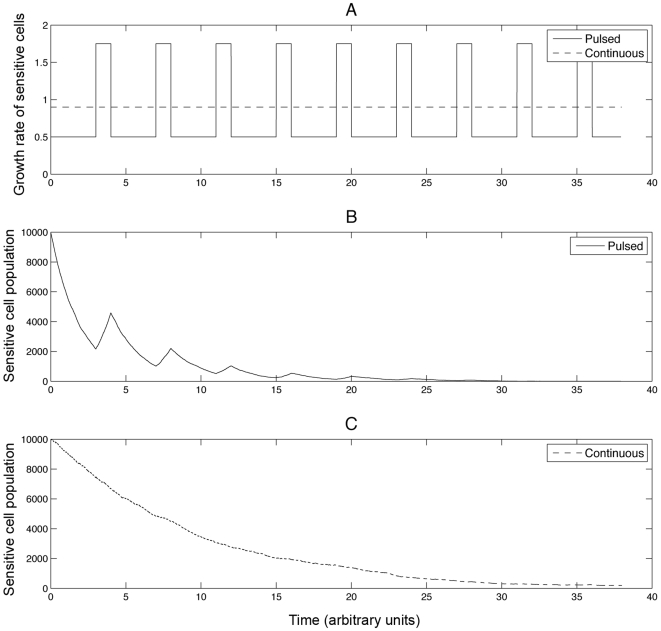
The dynamics of sensitive cancer cells during continuous and pulsed anti-cancer therapy. Subfigure (A) shows the dosing schedule specified by the growth rate of sensitive cancer cells as a function of time. The dashed line represents a dosing schedule in which the drug is administered at the maximum dose tolerated without treatment breaks. The solid line represents a schedule in which the drug is administered in pulses followed by drug holidays. Subfigures (B) and (C) show sample paths for the sensitive cell population during pulsed and continuous therapy, respectively. During treatment pulses as well as during continuous therapy, the sensitive cancer cell population declines while it expands during treatment breaks. Parameters are *M* = 10000, *r*_1_ = 1.75, *d*_1_ = 1.0, *c*_1_ = 1.0, *T_on_* = 3.0, and *T_off_* = 1.0.

Let us now consider the evolution of these cell populations during general pulsed treatment schedules. To investigate dosing strategies consisting of drug pulses and treatment holidays, we implement time-varying growth and death rates in the stochastic process model. During treatment pulses, the growth rates of sensitive and resistant cells are again denoted by 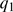 and 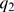, respectively, while their death rates are given by 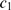 and 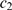. During drug holidays, the growth rates of sensitive and resistant cells are given by 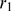 and 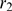, respectively, and their death rates by 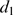 and 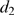 (see Table 1 for a summary of notation). Here we assume that the drug reaches its maximum concentration immediately after administration and remains at that level until the beginning of the treatment break; additionally, we assume that the intensity of the dose does not vary from pulse to pulse. The length of each treatment pulse is denoted as 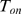 and the length of each treatment break is denoted as 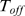. Each treatment cycle consists of one pulse and one break, and every treatment cycle has the same length: 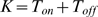. Thus, a strategy in which 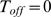 corresponds to the continuous dosing regimen. Both sensitive and resistant cells expand in the absence of therapy, 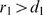 and 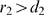. We consider only drugs at concentrations that lead to a reduction of the sensitive cancer cell population (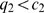). The (epi)genetic alteration conferring resistance may be neutral (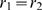), advantageous (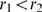), or disadvantageous (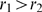) in the absence of therapy. If 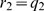 and 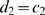, then the drug has no effect on resistant cancer cells and the mutation confers complete resistance; it is this case that we consider first. We investigate only viable treatment options (i.e. dosing strategies that are capable of depleting the sensitive cancer cell population). Specifically, we define 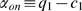, 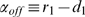, and 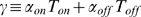, and require that the relation 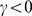 must hold. This criterion ensures that the sensitive cell population decreases on average over time. If this criterion is not met, the question of resistance becomes less important as the treatment schedule does not prevent the expansion of the sensitive cancer cell population. Figure 1 shows an example of the dynamics of sensitive cancer cells during continuous and pulsed drug administration.

**Table 1:** Notation.

Symbol	Definition (units, where applicable)
*X_t_*	Number of sensitive cancer cells at time *t*
*Y_t_*	Number of resistant cancer cells at time *t*
*M*	Initial number of sensitive cancer cells
*q*_1_	Growth rate of sensitive cancer cells during therapy (time ^−1^)
*c*_1_	Death rate of sensitive cancer cells during therapy (time ^−1^)
*q*_2_	Growth rate of resistant cancer cells during therapy (time ^−1^)
*c*_2_	Death rate of resistant cancer cells during therapy (time ^−1^)
*r*_1_	Growth rate of sensitive cancer cells during treatment break (time ^−1^)
*d*_1_	Death rate of sensitive cancer cells during treatment break (time ^−1^)
*r*_2_	Growth rate of resistant cancer cells during treatment break (time ^−1^)
*d_t_*	Death rate of resistant cancer cells during treatment break (time ^−1^)
*T_on_*	Length of treatment pulse (time)
*T_off_*	Length of treatment break (time)
*u*	Mutation rate per sensitive cell division
*p_i_*_,0_	Number of sensitive cancer cells at beginning of *i*-th treatment cycle
*p_i_*_,1_	Number of sensitive cancer cells at end of *i*-th treatment cycle
*B*	Number of sensitive cell divisions before extinction of sensitive cell population
*W*	Number of treatment cycles before extinction of sensitive cell population
*W_t_*	Number of treatment cycles until time *t*
*P*	Probability of resistance
*R(T)*	Expected number of resistant cells at time *T*
*T^i^_j_*	Example toxicity constraint (*i*-th member of *j*-th family)
*K*	*T_on_* + *T_off_*
*α_on_*	*q*_1_−*c*_1_
*α_off_*	*r*_1_−*d*_1_
*γ*	*α_on_T_on_* + *α_off_T_off_*

This table shows frequently used notation.

### The probability of resistance

Let us now calculate the probability of resistance during a given dosing schedule. Under the assumption of complete resistance, the probability of extinction of a resistant cell clone starting from one resistant cell is 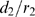, regardless of which dosing strategy is used [33]. The number of resistant cells produced from the sensitive cell population is on average proportional to the number of sensitive cell divisions; these two quantities are related through the mutation rate 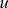.

As a preliminary calculation, consider the behavior of the sensitive cell population, 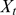, with constant growth rate 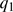 and death rate 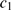. Under the assumption that the mutation rate 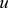 is small enough such that the stochastic emergence of resistant cells from sensitive cells has negligible effects on the sensitive cell number, we approximate 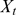 as a simple birth-death process. Recall that the initial size of the sensitive cell population is 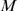; then the mean abundance of the sensitive cell population at time 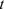 is approximated as 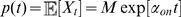. The number of sensitive cell divisions in the time interval 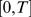 is approximately given by
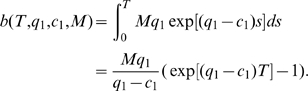
(1)The number of surviving resistant cell clones arising from the sensitive cell population in the time interval 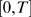 is then binomially distributed as 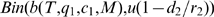.

Let us now study the probability of resistance under a general pulsed treatment regimen. Define 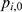 as the expected number of sensitive cancer cells at the beginning of the 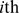 treatment cycle, and 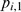 as the expected number of sensitive cancer cells at the beginning of the 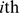 treatment holiday. Then we have 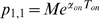, 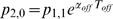, 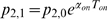, etc. We obtain the general formulae for the number of sensitive cancer cells at the beginning and end of the 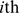 treatment cycle as
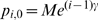
(2)

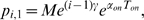
where 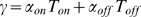. The number of cycles 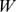 before extinction of the sensitive cell population is approximated as 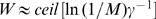. To estimate the total number of sensitive cancer cell births before extinction, we sum the number of births during the on- and off-treatment phases over all cycles. Let 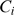 be the number of sensitive cancer cell births during the 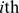 on-treatment phase and 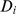 the number of births during the 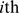 off-treatment phase. The expected number of births, 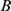, during the entire treatment regime is then approximated as
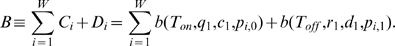
(3)Here 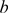 is the estimated number of sensitive cancer cell divisions in the treatment interval, evaluated as in equation (1). Next, let us define the functions 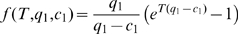 and 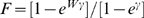. We can express each sum as the geometric series
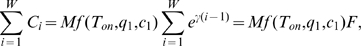
(4)

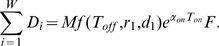



Then we obtain the expected number of sensitive cancer cell births during the entire duration of therapy as 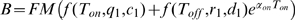. We can approximate 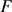 with 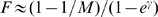. Substituting in the correct expressions for 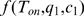 and 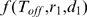, we obtain a final estimate for the number of sensitive cell divisions before the extinction of sensitive cells as
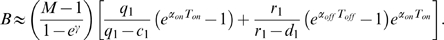



The number of surviving resistant cell clones produced from the sensitive cell population is a random variable with distribution 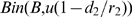. We can thus make a Poisson approximation to estimate the probability that at least one surviving resistant cell clone is produced before the extinction of sensitive cells as
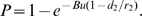
(5)


In the special case of continuous dosing, 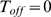, the number of sensitive cell divisions is approximated by
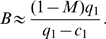
(6)As a consistency check, this formula can also be arrived at via equation (1) with the initial size of the sensitive cancer cell population, 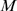, and the amount of time until the extinction of the sensitive cells, 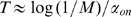. Then the probability of resistance emerging during continuous dosing is again calculated using formula (5) with equation (6).

When the (epi)genetic alteration confers partial resistance to the cell (i.e. when 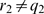 and/or 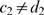), then the probability of resistance emerging during continuous dosing is given by
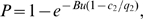
(7)where 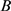 is again calculated as in equation (6). To accommodate this modification in pulsed schedules, we introduce ‘effective’ growth and death rates for resistant cells. The effective growth rate of resistant cells is given by 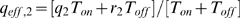, while the effective death rate of resistant cells is 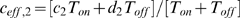. For general pulsed schedules, the probability of resistance is then approximated by
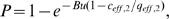
(8)where 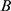 is calculated as in equation (5).

### The expected number of resistant cells

We next approximate the expected number of resistant cancer cells at time 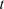. To calculate this quantity, we estimate the number of surviving resistant cell clones produced during each small time interval and then calculate the growth of each resistant cell clone until time 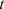. More precisely, we take the convolution of the rate of production of resistant cell clones from the sensitive cell process with the average rate of clonal expansion of resistant cells.

Let us first consider general pulsed treatment schedules. Using methods from the previous section, we find that the expected number of sensitive cancer cell divisions until time 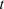 is given by
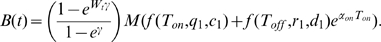
Here 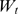 denotes the fractional number of treatment cycles until time 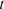. After making the approximation 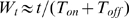, we have
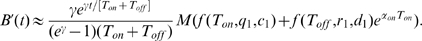
(9)Since the number of resistant cells produced directly from the sensitive cell population until time 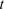 is binomially distributed, 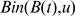, the expected number of such cells is given by 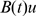. Thus we estimate the average number of resistant cells at time 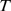 as
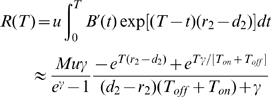
(10)

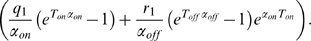



In the special case of continuous dosing strategies, 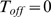, the average number of resistant cells at time 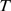 is given by
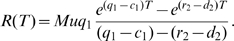
(11)Once again we can check for consistency by deriving this formula via equation (1). Recall that the expected number of sensitive cancer cell births starting from a population of size 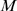 until time 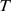 is given by 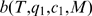 (equation (1)). Then the expected number of resistant cells produced is 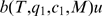, and the expected number of resistant cells at time 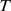 is given by
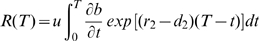


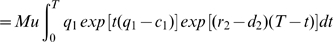


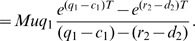



We are also interested in calculating the expected number of resistant cells averaged only over those patients who develop resistance. This quantity is clinically relevant since many treatment choices may inevitably lead to resistance; in those cases, the drug should be dosed in such a way that the number of resistant cells is minimum, thereby maximizing the time until detection of resistance and disease progression. Mathematically, this amounts to estimating the expected size of the resistant cell population, conditioned on the event that at least one surviving resistant clone is produced prior to the extinction of sensitive cancer cells. We make the approximation that the expected resistant cell number, conditioned on the complementary event of no surviving resistant cell clones, is negligible. Then the expected number of resistant cells averaged over the cohort of patients who develop resistance is estimated as
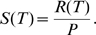
(12)


### Pre-existing resistance

Suppose that at the start of therapy there exists a small population of resistant cells. We may then adapt the theory to calculate the probability of resistance and expected size of the resistant clone under various dosing schedules. Let us consider the initial population as two separate populations: 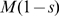 sensitive cells and 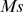 resistant cells, where 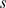 is the initial fraction of resistant cells (assume for simplicity that 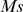 is an integer). Then the probability of avoiding resistance is given by the probability that the pre-existing resistant cell clones become extinct times the probability that the initial sensitive cell population does not give rise to any surviving resistant clones during treatment. Let 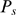 denote the probability, calculated as in equation (5), of *de novo* resistance arising from the initial sensitive population of size 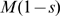. The probability of extinction of the pre-existing clone is given by 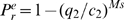 if 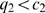 and 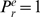 otherwise. (Note that 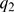 and 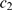 may be replaced by 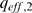 and 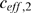 in the case of pulsed schedules with partial resistance). Then the total probability of resistance is given by
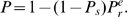
(13)Let 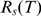 represent the expected number of resistant cells arising from the initial sensitive cell population of size 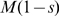, calculated as in equation (10). The expected number of resistant cells at time 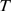 is given by 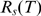 plus the expected current size of the initially resistant population. Thus we have
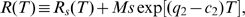
(14)where once again the rates 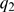 and 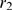 may be replaced by their effective values in the case of pulsed therapy with partial resistance.

## Results

Exact stochastic simulations of the process 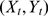 are performed using the standard Monte Carlo technique; each time an event occurs, a cell is chosen to divide or die based on the current cell growth and death rates and the population size of each cell type. When the drug concentration changes, the cell growth and death rates are modified accordingly.

**Figure 2:**
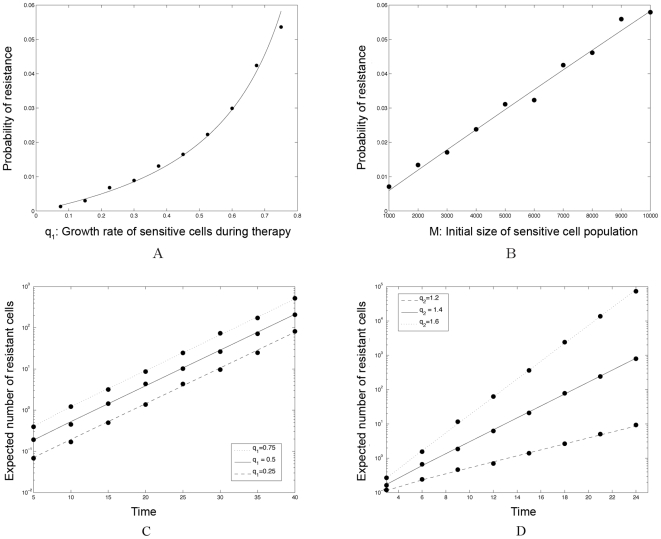
The probability of resistance and expected number of resistant cells during continuous therapy. (A) The probability of developing resistance during continuous therapy as a function of the growth rate of sensitive cells during treatment, *q*_1_. The probability of resistance increases with the growth rate. Solid lines represent the analytical approximations and Monte Carlo simulation results are plotted as dots. Parameters are *M* = 10000, *q_2_* = 1.25, u = 10^−5^, and *c*_1_ = *c*_2_ = 1.0. (B) We show the probability of developing resistance during continuous therapy as a function of the number of sensitive cancer cells at diagnosis, *M*. The probability of resistance increases linearly with *M*. Parameters are *q*_1_ = 0.75 and all others as in (A). (C) We show the expected number of resistant cells as a function of time during continuous therapy for different values of the growth rate of sensitive cells during treatment, *q*_1_. The expected number of resistant cells increases with time and with *q*_1_. Parameters are *M* = 1000, *q*_2_ = 1.2, *u* = 10^−4^, and *c*_1_ = c_2_ = 1.0. (D) We show the expected number of resistant cells as a function of time during continuous therapy for different values of the growth rate of resistant cells during treatment, *q*_2_. The expected number of resistant cells increases with time and with *q*_2_. Parameters are *q*_1_ = 0.5 and all others as in (C).

Let us now investigate the fit between the analytic approximations and exact stochastic computer simulations, as well as the parameter dependence of these approximations. We first study the dependence of the probability of resistance emerging during a continuous dosing schedule on the growth rate of sensitive cancer cells during therapy, 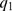 (Fig. 2A), and their initial number, 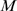 (Fig. 2B). The simulations exhibit a good fit with the analytical approximations. As the growth rate of sensitive cells during therapy (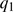) increases, the risk of developing resistance increases as well. Similarly, as the initial size of the sensitive cancer cell population increases, the number of sensitive cell divisions until extinction becomes larger, thus enhancing the likelihood of producing a successful resistant cell clone. Hence the probability of developing resistance also increases with 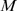.

We next investigate the expected number of resistant cells as a function of time for varying growth rates of sensitive and resistant cells during continuous treatment. When the sensitive cell growth rate during treatment, 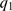, increases and the growth rate of resistant cells, 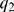, is kept constant, then the expected number of resistant cells increases (Fig. 2C). This behavior is apparent from equation (11); during therapy the denominator 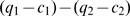 is always negative, thus making 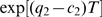 the dominant term in the numerator. Therefore the growth rate of the expected population size is dominated by the growth rate of resistant cells at later times as the other time-dependent term in the numerator approaches zero. Figure 2D confirms that as 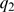 increases, the expected number of the resistant cell population also increases.

**Figure 3:**
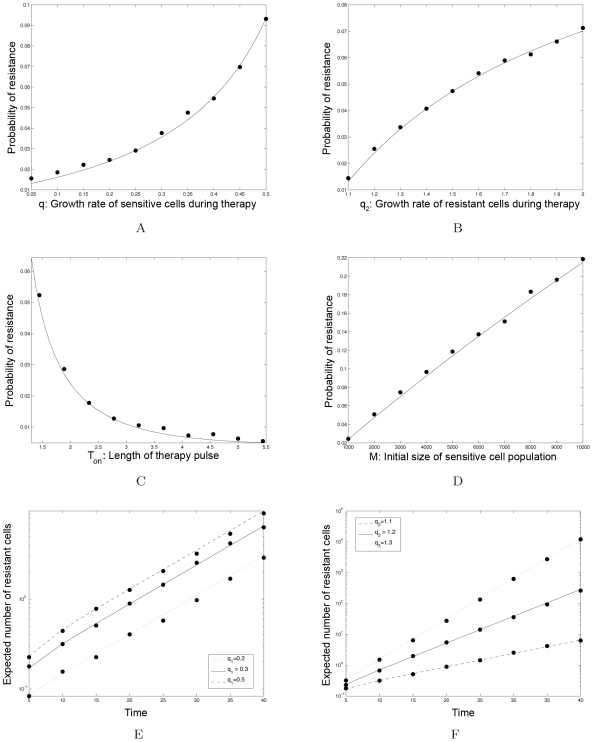
The probability of resistance and expected number of resistant cells during pulsed therapy. (A) We show the probability of resistance during pulsed therapy as a function of the growth rate of sensitive cancer cells during each treatment phase, *q*_1_. The risk of resistance increases as the growth rate is enhanced. Lines represent the analytical predictions and dots show the Monte Carlo simulation results. Parameters are *M* = 1000, *r*_1_ = 1.3, *r*_2_ = *q*_2_ = 1.1, *u* = 10^−4^, and *d*_1_ = *d*_2_ = *c*_1_ = *c*_2_ = 1.0. (B) We show the probability of resistance during pulsed therapy as a function of the growth rate of resistant cancer cells during a treatment phase, *q*_2_. The risk of resistance increases as the growth rate is enhanced. Parameters are *q*_1_ = 0.5 and all others as in (A). (C) We show the probability of resistance during pulsed therapy as a function of the duration of each treatment phase, *T_on_*. The risk of resistance decreases as the pulse becomes longer. Parameters are as above. (D) We show the probability of resistance during pulsed therapy as a function of the initial size of the sensitive cancer cell population, *M*. The risk of resistance increases with *M*. Parameters are as above. (E) and (F) We show the expected number of resistant cells as a function of time during pulsed therapy when *q*_1_, (E), and *q*_2_, (F), are varied. The expected number of resistant cells increases with both quantities. Parameters are as above.

Let us now consider treatment schedules incorporating pulsed doses and treatment holidays. We first investigate the dependence of the probability of resistance on the growth rates of sensitive and resistant cancer cells during treatment phases, 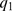 and 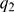, the duration of each treatment pulse, 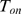, and the initial number of sensitive cancer cells, 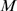 (Figs. 3A–D). As in the case of continuous dosing, the probability of resistance increases as 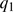 and 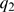 increase. An increase in the duration of each treatment pulse, 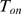, decreases the probability of resistance while an increase in 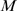 linearly enhances the risk of resistance. The parameter dependence of the expected number of resistant cells as a function of time is shown in Figs. 3E and 3F. The expected number of resistant cells increases with increasing growth rates of sensitive and resistant cancer cells during therapy (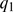 and 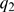).

### Optimizing dosing strategies

Using the estimates derived above, we now propose a method for optimizing dosing strategies to minimize the probability of resistance. In cases where the emergence of resistance is certain, this method will predict a dosing strategy that maximally delays the detection of resistance by minimizing the number of resistant cancer cells. The optimal dosing strategy is selected from a range of tolerated treatment schedules specified by toxicity constraints. In practice, these toxicity constraints, in addition to the growth and death rates of sensitive and resistant cells at varying dose levels, must be determined experimentally for each drug and cancer type. In the following we will construct example toxicity constraints to demonstrate the methodology and test for sensitivity to the constraint profile.

**Figure 4:**
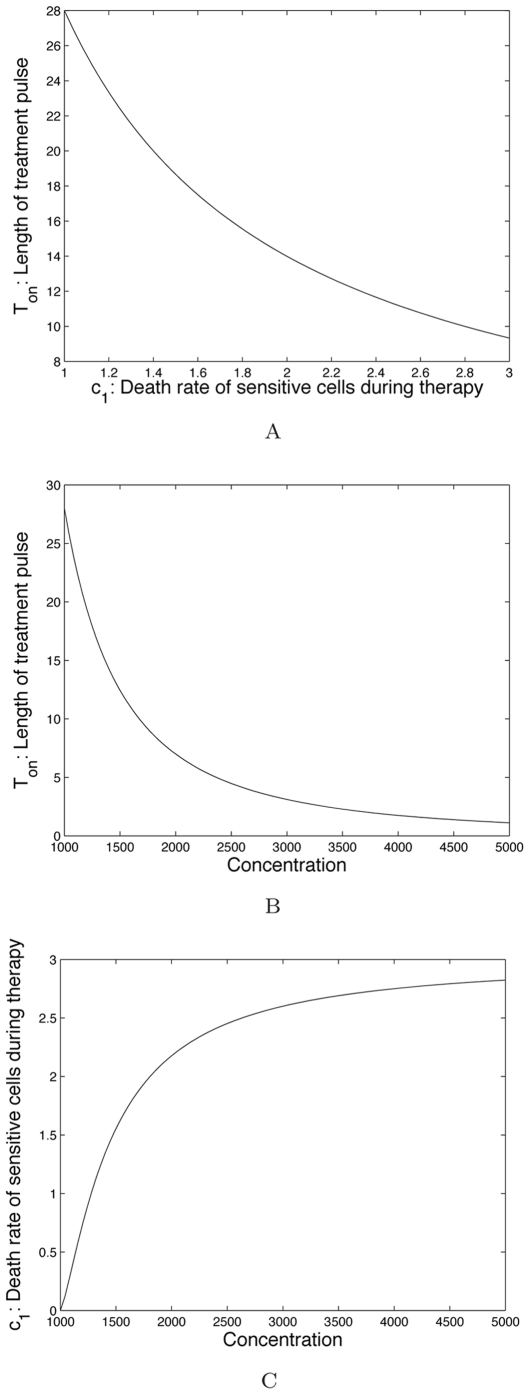
Examples for toxicity constraints. Subfigure (A) shows an example toxicity constraint in the form of a function defining the maximum tolerated duration of treatment, *T_on_*, versus the death rate of sensitive cancer cells during therapy, *c*_1_, for a 28-day treatment cycle. This type of toxicity constraint can be derived from experimental and clinical data in the form shown in subfigures (B) and (C). In subfigure (B), we display an example of the type of data available from clinical trials that relate *T_on_* to the drug concentration. Hence for a given drug concentration, a treatment pulse of *T_on_* days was tolerated. In (C), we show an example of how the drug concentration can be related to the death rate of sensitive cancer cells during treatment, *c*_1_, through *in vitro* experiments.

A modification of treatment schedules can change the duration of each treatment pulse (affecting 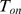 and 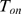), the intensity of the dose (affecting growth and death rates of sensitive cancer cells, 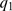 and 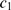), or both. When considering complete resistance, the growth and death rates of resistant cells are unaltered by changing treatment strategies. We assume that all other parameters are unaffected by changes in administration schedules as well. Thus, we consider toxicity constraints to provide a bounded domain in the four-dimensional parameter space spanning 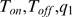, and 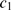. We can immediately reduce the dimension of the constraint domain to two, since 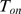 specifies 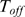 explicitly through the fixed length of the treatment-and-break cycle, 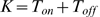, and 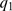 and 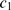 are both dependent on the concentration of the drug and thus cannot vary independently. Therefore, we consider toxicity constraints in the form of a function specifying the maximum amount of time, 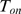, that a drug can be administered to a patient at a particular concentration before causing dose-limiting toxicities. In the following, we make the simplifying assumption that this drug concentration specifies the death rate of sensitive cancer cells, 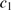, and does not alter the growth rate, 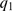; alternatively, we can also investigate treatment strategies that modulate the growth rate rather than the death rate of sensitive cancer cells, or both. We assume that such relationships between 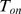 and 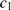 are monotonically decreasing functions of 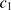; see Figure 4A for an example of a toxicity constraint.

From clinical trial data we obtain the maximum amount of time for which a range of drug concentrations are tolerated, leading to a relationship between 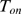 and the drug concentration. The effect of particular drug concentrations on 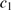 and/or 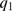 may then be found experimentally by exposing sensitive cancer cells to drug doses and measuring the growth and death rates 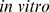. Such investigations identify a toxicity constraint relating 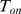 and 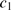. We display example constraint functions in Figures 4B and 4C.

**Figure 5:**
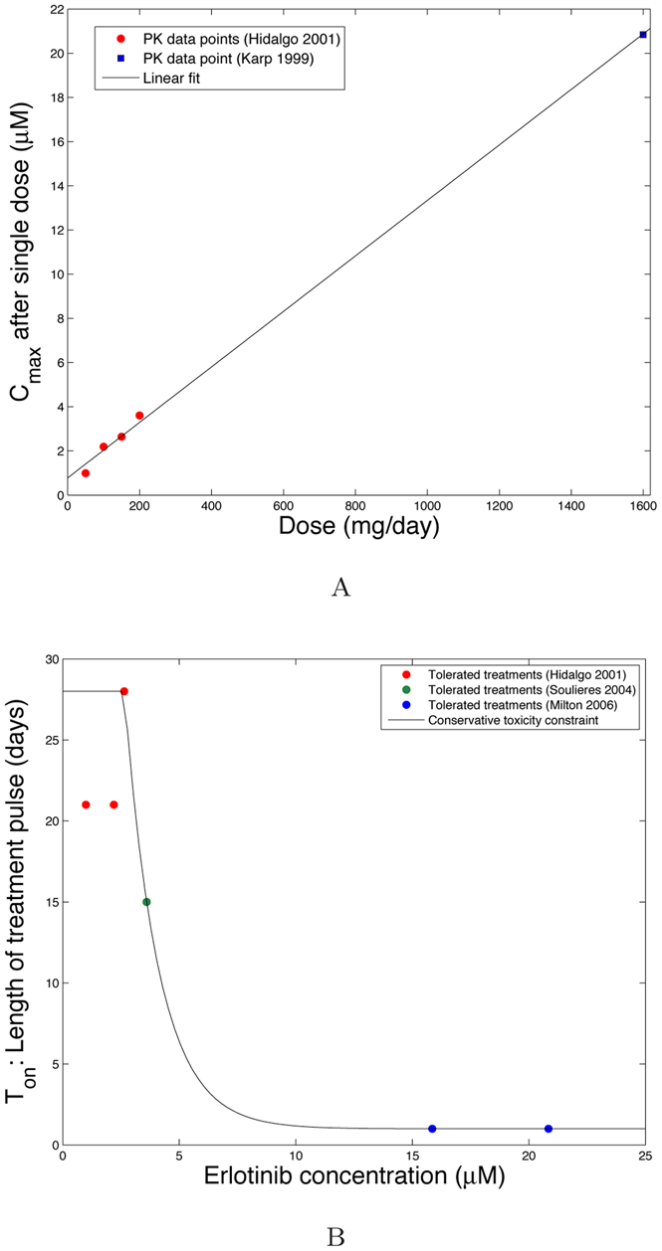
Erlotinib pharmacokinetic data and toxicity constraint. Subfigure (A) shows the relationship between dose and plasma *C_max_* concentration obtained after a single dose (*µM*). These data were reported in [34],[36]. Subfigure (B) shows points depicting clinically tolerated schedules, as reported in [34]–[36]. The doses administered are converted to plasma concentration using the linear relationship found in subfigure (A). We also plot a conservative toxicity constraint where we assume that any concentration or length of pulse increased to levels beyond those tolerated in the trials would not be admissible.

We next show some example toxicity data for the targeted drug erlotinib, which is an EGFR inhibitor used in treating solid malignancies such as non-small cell lung cancer. Compiling data from several clinical trials [34]–[36], we obtain a relationship between the drug dose and plasma concentration (measured as the maximum concentration achieved after a single dose). This data is plotted in Figure 5A; here we observe a relatively linear relationship between dose and plasma concentration. We also compiled data points on the number of days each particular dose was tolerated in continuous daily administration. We converted each dose level to concentration using the linear relationship found, and plot these points in Figure 5B. A conservative toxicity constraint in terms of 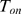 vs. concentration is plotted, where we assume that any concentration or length of pulse increased beyond what was tolerated in the trials would not be admissible. This toxicity constraint, in conjunction with further experimental data on the growth and death rates of sensitive and resistant cancer cells at various concentrations, would enable us to calculate optimal dosing schedules for this specific system using our model.

For our theoretical investigations, we now introduce several example families of toxicity constraints to test for sensitivity of the probability of resistance to several key aspects of the shape of the curve. All of these example constraints are convex, monotonically decreasing functions of 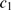. Thus we have implicitly assumed that as the drug concentration increases and the cell death rate increases, the maximum tolerated length of a treatment pulse decreases. In the first family, we vary the maximum dose that can be tolerated for the full treatment schedule of 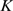 days. In the second family we vary the maximum dose that can be tolerated for just one day, and in the third family we vary the degree of convexity of the constraint curve, or the initial rate of decrease in 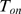 as the concentration increases.

**Figure 6:**
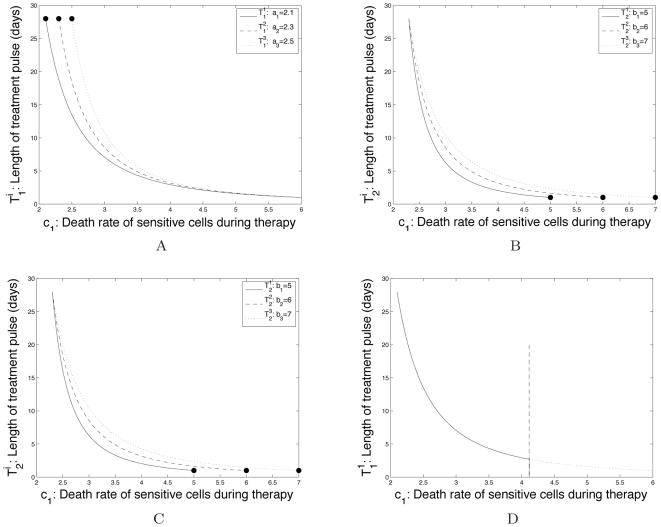
Families of toxicity constraints relating the duration of a treatment phase to the death rate of sensitive cancer cells. (A) We show examples for toxicity constraints which vary the endpoint representing the maximum dose that can be tolerated for a full treatment cycle of *K* = *T_on_* days. The treatment cycle is *K* = 28 days, and the endpoint representing the maximum dose that is tolerated for just one day is specified by the death rate *c*_1_ = 6. Black dots represent different continuous-dose endpoints. (B) We show examples for toxicity constraints which vary the endpoint representing the maximum dose that can be tolerated for a single day only, *T_on_* = 1. Parameters are as above and black points represent the high-dose endpoints. (C) We show examples for toxicity constraints which vary the degree of the convexity of the curve. Parameters as above. (D) We show an example for a toxicity constraint (equation (16)) in which the range of *c*_1_ is restricted to satisfy the viable treatment option constraint, *γ*<0. The dotted line shows the full toxicity constraint curve, *T_1_^1^*, while the solid line shows the portion of the curve in the range in which *γ*<0. Parameters are *r*_1_ = 1.3, *d*_1_ = 1, and *q*_1_ = 1.3.

Consider the first family of toxicity curves in Figure 6A, specified by
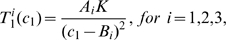
(15)where 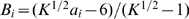 for 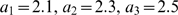 and 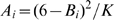. In our notation, 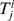, the subscript 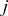 denotes the constraint family and the superscript 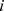 indicates a specific function belonging to this family. These constraints serve to vary the endpoint representing the maximum dose that can be tolerated for a full treatment cycle (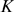 days) while fixing the endpoint representing the maximum dose that is tolerated for just one day of a treatment cycle, specified by the death rate 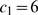. In other words, in this family of constraints we vary the continuous-dose concentration endpoint (represented by black circles in Figure 6A) of the toxicity constraint via the parameter 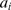, while keeping the form of the constraint and the high-dose concentration endpoint fixed.

We also test for sensitivity to two other aspects of the toxicity constraints: the high-dose concentration endpoint (i.e. the maximum dose that is tolerated for just one day) and the degree of concavity of the curve. Figure 6B shows a family of constraints varying the high-dose endpoints (shown in black circles). These example constraints are specified by equation
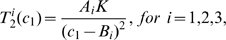
(16)where 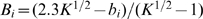 for 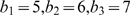 and 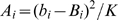. Likewise, a family of constraints varying the degree of convexity is exhibited in Figure 6C and specified by the following equations:
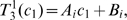
(17)

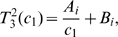


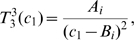
where, for each function, the 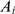 and 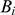 are determined by setting the endpoints to be
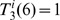
(18)

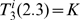



Once the toxicity constraint is established (e.g. Figure 4), the tolerable range of treatment schedules is specified by the area under the curve on the 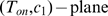. We then aim to locate the optimal point within this area that minimizes the probability of resistance. In situations in which the optimum probability of resistance is 1 or close to 1, we aim to locate the optimal point minimizing the expected number of resistant cells conditional on developing resistance, thus maximizing the time until disease progression. We note from the analytical approximations that a change in the mutation rate 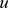 does not modify the choice of optimal dosing schedule. We also observe from our analytical approximations that the optimizing points must always lie directly on the toxicity constraint curve itself – intuitively, any point lying below the toxicity curve represents a weaker than tolerated dosing schedule and hence cannot minimize the risk of resistance. Once a minimizing point is located, the optimal treatment schedule is entirely specified since the duration of treatment pulses are given by 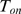, the length of the drug holiday is given as the remainder of the cycle duration 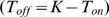, and the intensity of the dose is specified by the death rate of sensitive cells, 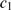.

To illustrate this concept, let us consider the toxicity constraint 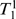 from equation (15). Recall the constraint 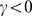 restricting the treatment schedules to viable dosing strategies in which the population of sensitive cells decreases overall in time. This constraint may restrict the domain of the toxicity curve to a limited range of 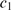. For the current example, this restriction is shown in Figure 5. We can then calculate the probability of resistance, the expected number of resistant cells, and the conditional expected number of resistant cells over the range of treatment schedules specified by this restricted constraint curve. Note again that in the formula describing the expected number of resistant cells, equation (10), the growth rate of 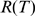 is dominated by the growth rate of resistant cells, 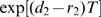, at later times, since the other time-dependent term in the expression, 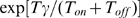, approaches zero as 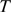 increases. Thus we can neglect the latter term when considering the long-term growth of the resistant cell population. Rewrite equation (10) as 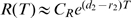. Here 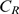 is the time-independent constant comprised of the remaining terms in equation (10) except for 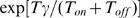. Analogously, the expected number of resistant cells conditional to the emergence of resistance is approximated by 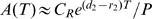.

**Figure 7:**
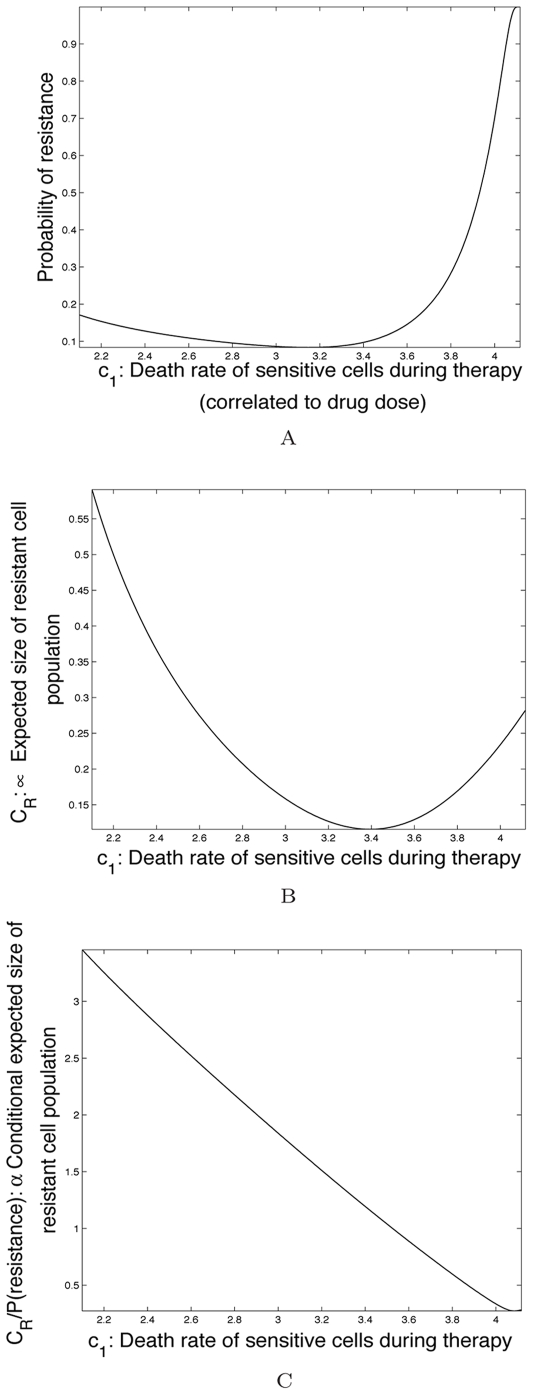
The identification of optimum dosing strategies. (A) We show the probability of resistance, *P*, over the range of treatment schedules specified by the restricted (solid) constraint curve from Figure 6(d). The probability of resistance is minimized when *c*_1_ = 3.15. We can then use the toxicity constraint curve to identify all other specifications of the dosing schedule, such as *T_on_*, *T_off_*, the concentration etc. Parameters are *r*_1_ = 1.3, *d*_1_ = 1, *q*_1_ = 1.3, *r*_2_ = 1.3, *d*_2_ = 1, *M* = 10^9^, and *u* = 5·10^−10^. (B) We show the time-independent term of the equation describing the number of resistant cells, *C_R_*, over the same constraint curve as above. This term is minimized when *c*_1_ = 3.4, which does not coincide with the optimum dosing strategy identified in (A). Therefore the strategy minimizing the probability of resistance may not coincide with the strategy maximizing the time until detection of resistant cells. (C) We show the value proportional to the conditional number of resistant cells, *C_R_/P*, over the same constraint curve. Parameters are as above.

In Figures 7A–C, we show the probability of resistance, 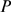, the time-independent term of the equation describing the number of resistant cells, 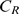, and 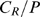 over the range of treatment schedules specified by the restricted constraint curve from Figure 6D. As the drug concentration and hence the death rate of sensitive cancer cells, 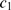, increase, we move along the constraint curve from the continuous-dose endpoint towards the high-dose endpoint. As a particular numerical example, consider an initial number of sensitive cancer cells of 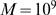, a mutation rate conferring resistance of 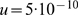, and a neutral resistance mutation (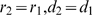). Then the probability of resistance, shown in Figure 7A, is minimized when 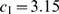. This result is subsequently used to identify the corresponding optimal treatment schedule in Figure 6D, which in this case is given by 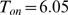 days, 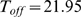 days, and a drug concentration achieving 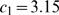. When this optimal treatment schedule is used, the probability of resistance is below 10%. However, if a higher dose is chosen, the probability of resistance may increase up to 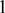. This example illustrates the importance of locating the optimal dosing regime for the clinical management of patients.

The values proportional to the expected number of resistant cells, 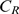, and to the conditional expected number of resistant cells, 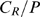, are displayed in Figures 7B and 7C. Interestingly, in the event that resistance occurs, the optimal treatment schedule for minimizing the resistant cell population is specified by 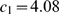, which differs from the optimal schedule for minimizing the probability of developing resistance. For a general cohort of patients treated with this dosing schedule, the probability of developing resistance would be close to 1; for the subset of patients who do develop resistance, however, this dosing schedule would delay disease progression by the largest amount of time.

### Parameter dependence of optimal dosing strategies

Let us now examine the dependence of these optimal dosing regimens on variations in parameters and toxicity constraints. Specifically, we investigate the sensitivity of the optimal dosing strategies to several characteristics of the toxicity curves: the maximum dose that can be administered for the whole treatment cycle of 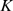 days (the continuous-dose endpoint), the maximum dose that can be administered for one day only (the high-dose endpoint), and the degree of concavity of the toxicity curve. The optimal dosing regimens are identified over a range of parameter values of 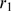 and 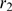.

**Figure 8:**
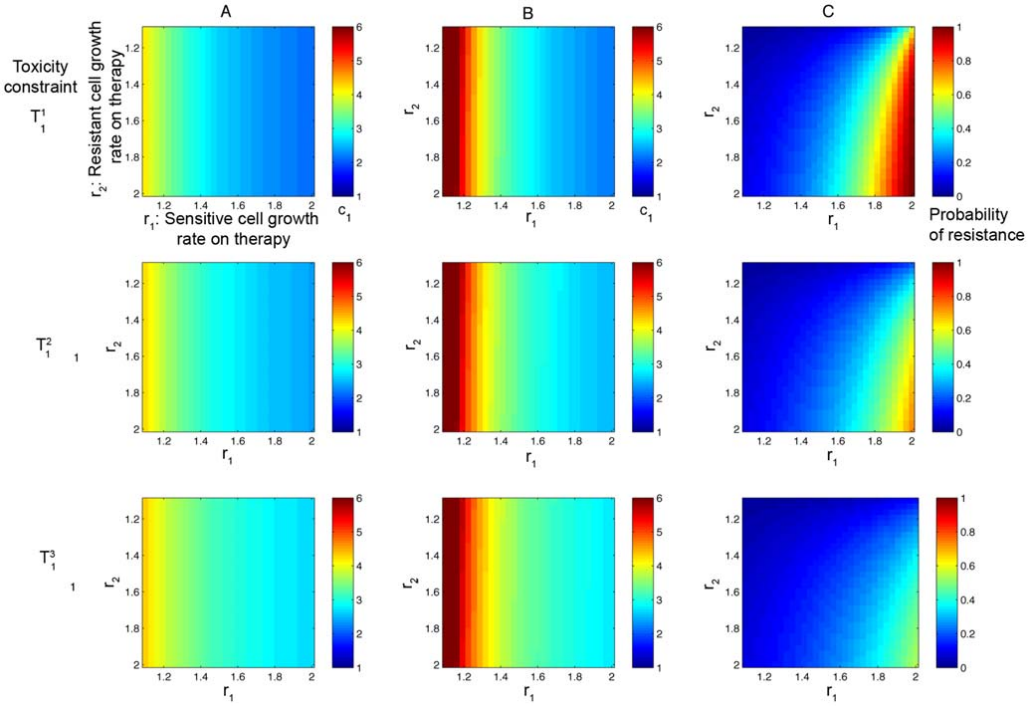
Optimal dosing regimens for the family of toxicity curves in **Figure 6(A)** **under variation of the continuous-dose endpoint.** In column (A), the color at each point represents the value of *c*_1_ that corresponds to the dosing schedule which minimizes the probability of resistance. In column (B), the color at each point represents the value of *c*_1_ that corresponds to the dosing schedule which minimizes the conditional expected number of resistant cells. In column (C), the minimal probability of resistance corresponding to the optimal schedule from column (A) is plotted. Rows 1,2, and 3 show results for constraints *T*_1_^1^, *T*_1_^2^, and *T*_1_^3^. Parameters are *d*_1_ = 1, *q*_1_ = 1.3, *M* = 10^9^, and *u* = 5·10^−10^.

First, we consider the family of curves 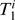 for 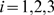 (equations (15) and (14) as shown in Figure 6A). The optimal dosing strategy minimizing the probability of resistance and/or the conditional number of resistant cells is displayed in Figure 8. In column (A), we show the value of 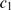 that corresponds to the dosing schedule which minimizes the probability of resistance for a given 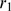 and 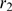. The corresponding minimal probability of resistance is shown in column (C). Column (B) displays the value of 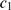 that specifies the dosing schedule minimizing the conditional expected number of resistant cells, i.e. maximizing the amount of time until disease progression in patients who develop resistance. The rows show the results for constraints 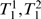, and 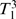, respectively. Note that the optimal dosing schedules in the first and second column are not identical, reflecting the fact that the recommended dosing regimens for these two clinical goals are different. In addition, we observe that as the continuous-dose endpoint is varied, the minimal probability of resistance changes (in column (C)) while the optimal dosing schedules remain relatively unchanged. In particular, the minimal probability of resistance decreases as the continuous-dose endpoint shifts to the right.

**Figure 9:**
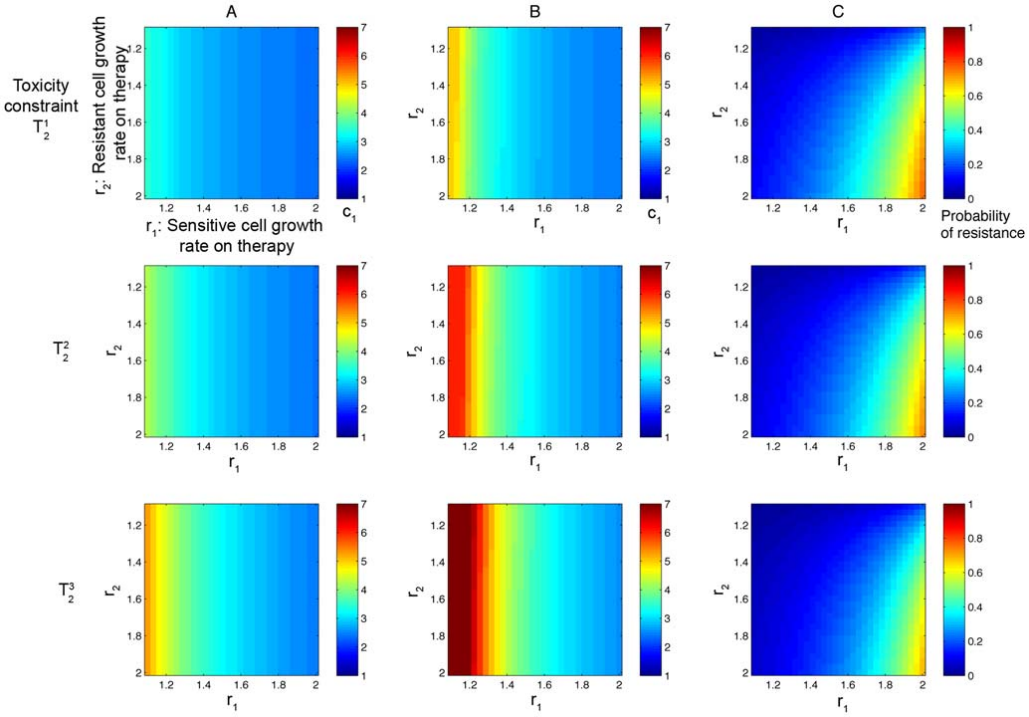
Optimal dosing regimens for the family of toxicity curves in **Figure 6(B)** **under variation of the high-dose endpoint.** In column (A), the color at each point represents the value of *c*_1_ that corresponds to the dosing schedule which minimizes the probability of resistance. In column (B), the color at each point represents the value of *c*_1_ that corresponds to the dosing schedule which minimizes the conditional expected number of resistant cells. In column (C), the minimal probability of resistance corresponding to the optimal schedule from column (A) is plotted. Rows 1,2, and 3 show results for constraints *T*_1_^1^, *T*_1_^2^, and *T*_1_^3^. Parameters are *d*_1_ = 1, *q*_1_ = 1.3, *M* = 10^9^, and *u* = 5·10^−10^.

Next we consider the family of curves 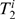, for 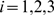 (equations (15) and (16), shown in Figure 6B). We plot the results in Figure 9, where the columns show the optimal treatment schedules and the probability of resistance for constraints 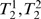, and 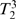, respectively. For both clinical goals of minimizing the probability of resistance and maximizing the time until detection of resistance, we observe that as the maximum dose tolerated for one day (the high-dose endpoint) is increased, the optimal dosing schedule shifts slightly to a more high-dose pulsed regimen in some regions of the parameter space (particularly when 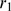 is small). However, the minimal probability of resistance changes only slightly as this endpoint is increased.

**Figure 10:**
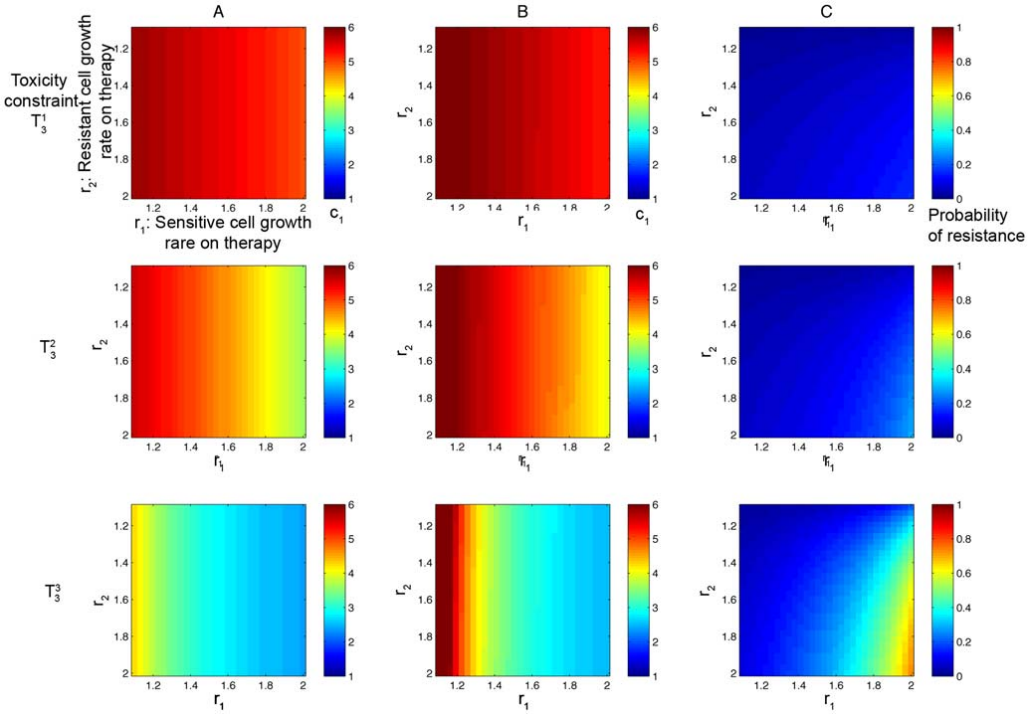
Optimal dosing regimens for the family of toxicity curves in **Figure 6(C)** **under variation of the convexity of the toxicity constraint.** In column (A), the color at each point represents the value of *c*_1_ that corresponds to the dosing schedule which minimizes the probability of resistance. In column (B), the color at each point represents the value of *c*_1_ that corresponds to the dosing schedule which minimizes the conditional expected number of resistant cells. In column (C), the minimal probability of resistance corresponding to the optimal schedule from column (A) is plotted. Rows 1,2, and 3 show results for constraints *T*_1_^1^, *T*_1_^2^, and *T*_1_^3^. Parameters are *d*_1_ = 1, *q*_1_ = 1.3, *M* = 10^9^, and *u* = 5·10^−10^.

Lastly, we consider the family of curves 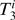, for 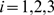 (equation (17), shown in Figure 6C). We plot the results in Figure 10. The columns again show the optimal schedules and the probability of resistance for constraints 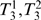, and 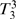. The results for the first two constraints, 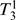 and 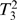, differ markedly from those of 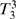. In particular, for functions with a lower degree of convexity, a high-dose pulsed treatment is optimal for both clinical goals. For these cases a minimal probability of resistance near zero can be achieved. However, for 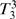 the optimal dosing schedule shifts more towards the continuous end of the dosing spectrum, and in certain parameter ranges the minimal probability of resistance reaches higher values.

### Non-cytoreductive therapies

So far we have only considered treatment strategies during which the total number of sensitive cancer cells declines on average, i.e. when 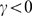 holds. However, for some therapies and cancer types it is impossible to reduce the number of sensitive cancer cells. Then the goal of therapy becomes to slow or even halt the rate of tumor growth. For these cases, the probability of resistance is always one. However, we can still identify treatment schedules that maximally delay progression of disease by controlling the number of resistant cells. The approximations for the expected number of resistant cells derived above remain valid, except when 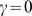. In this case, we revisit the calculation of 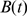 and estimate the total number of births during on- and off-treatment phases as
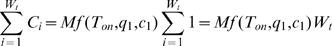
(19)

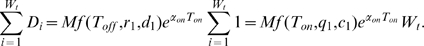
Once again making the approximation 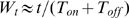, we obtain
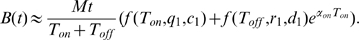
After taking the convolution of the derivative 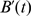 with the expected growth rate of resistant cells, we obtain the expected number of resistant cells at time 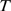 as
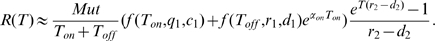
Note that for cases when 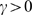, the formula for the expected number of resistant cells, equation (10), experiences a singularity in the denominator when 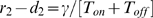, i.e. when the net growth rate of the resistant cancer cells equals the net growth rate of the sensitive cancer cells. However, the range of therapies considered should be restricted to those in which the net growth rate of sensitive cancer cells is less than that of resistant cancer cells; otherwise, the problem of resistance is secondary to the problem of controlling the sensitive cell population. In these cases, the singularity does not occur.

## Discussion

In this paper, we have constructed a simple mathematical model using birth and death processes to describe the evolution of resistance during targeted anti-cancer therapy. We have derived and validated analytical approximations to this model, which provide a useful tool for predicting the risk of resistance and the growth of resistant cell populations under various dosing strategies. We have used our model and estimates to develop a methodology for designing optimal drug administration strategies to minimize the risk of resistance. In cases in which the risk of resistance is high for any treatment schedule, these strategies are modified to maximize the time until the progression of disease.

The probability of resistance is shown to be largely dependent on 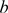, the rate of sensitive cell division, which is the product of the current sensitive cell population size and its growth rate. Drugs whose main goal is to increase the death rate of sensitive cells can decrease the sensitive cell population, thus decreasing 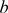 and reducing the probability of resistance; however, if the initial tumor size is large, it may take a significant amount of time to deplete the sensitive cell population. During this delay, there is still a high probability of generating resistant mutants since the sensitive cell proliferation rate is unchanged. On the other hand, for drugs that inhibit sensitive cell proliferation and effectively reduce the growth rate of sensitive cells, the quantity 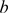 is immediately reduced to zero regardless of the initial size of the tumor. This implies that drugs that inhibit cancer cell proliferation could be promising for the prevention of resistance in the absence of pre-existing resistant cell clones. Combination therapies in which an inhibitor of sensitive cell proliferation is dosed continuously while short, high pulses of a drug that increases the death rate of resistant cells are administered may also be of interest, as are any combination strategies which separately target the sensitive and resistant populations.

We have also extended the theory to incorporate pre-existing resistant cells at the start of therapy. The effect of pre-existing resistant clones on the optimal dosing strategy is highly dependent upon system parameters including the growth and death rates of sensitive and resistant cells, the initial tumor size, and initial number of resistant cells. Consider the probability of resistance in this scenario, given by equation (13). We note that the term 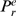, denoting the probability of extinction of the pre-existing clone, consists of the 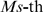 power of a quantity usually less than one 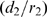. Thus even a small population of pre-existing resistant cells can cause the total probability of resistance to be effectively equal to one. For example, if the growth rate of resistant cells is twice their death rate, then the probability of extinction for an initial population consisting of only 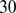 resistant cells evaluates to 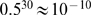. Then the total probability of resistance, given by equation (13), is approximately one. Therefore, the presence of even a small number of resistant cells at the start of therapy can effectively prevent a cure. In these cases, we may instead attempt to delay disease progression by controlling the number of resistant cells. Equation (14) describes the current size of the resistant population as the sum of the average *de novo* and pre-existing resistant clone sizes. Observe that both terms in this expression grow at the same exponential rate; the term for pre-existing resistance starts at time zero with the value 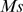, while the term for *de novo* resistance starts with value zero at time zero. This fact has implications for treatment schedules in the case of pre-existing resistance: as long as an eventual decline of sensitive cancer cells is achieved, high-dose strategies which slow the effective net growth rate of resistant cells may be more effective than low-dose strategies aimed at maximal continuous inhibition of sensitive cells.

By testing several families of toxicity constraints, we have observed that the optimal dosing strategies are strongly affected by the degree of convexity of the toxicity curve, thus delineating a clear priority in experimental efforts to determine the parameters of this constraint. In our experience of studying published results of Phase I clinical trials of molecularly targeted anti-cancer therapies, patient toxicity reports are usually not detailed enough to accurately determine toxicity curves. In light of our observations, we would like to stress the importance of publishing detailed quantitative data on toxicity in clinical trials, so that statistical analyses can be performed to inform these constraint curves. It is also important to estimate the growth and death rates of sensitive and resistant cancer cells during administration of diverse drug concentrations. These curves can be estimated by studying the growth and death kinetics of cancer cells, either *in vivo* or *in vitro*. For example, *in vitro* net growth rates can be determined by subjecting sensitive and resistant cell populations to drug at varying concentrations and counting viable cells at multiple time points. Then, through fluorescence-activated cell sorting techniques, the amount of cell death at multiple time points can be observed, providing the cell death rate at each drug concentration. If the parameters of the model are also estimated for treatment with conventional cytotoxic chemotherapeutics, then our model can be applied to these treatment choices as well. This methodology, together with key parameters derived experimentally, can aid in the design of optimum administration strategies of treatment options for all cancer types that evolve resistance via a single (epi)genetic alteration.
