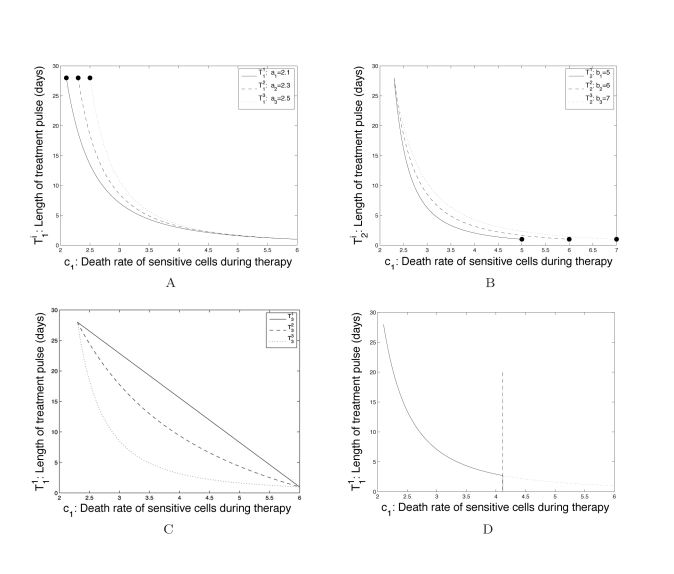# Correction: Evolution of Resistance to Targeted Anti-Cancer Therapies during Continuous and Pulsed Administration Strategies

**DOI:** 10.1371/annotation/d5844bf3-a6ed-4221-a7ba-02503405cd5e

**Published:** 2009-12-23

**Authors:** Jasmine Foo, Franziska Michor

In Figure 6, panel C is a duplicate of panel B. The correct Figure 6 can be viewed here: 

**Figure:**